# Health inequities in Bhutan's free healthcare system: a health policy dialogue summary

**DOI:** 10.1002/puh2.34

**Published:** 2022-11-02

**Authors:** Karma Tenzin, Thinley Dorji, Gampo Dorji, Don Eliseo Lucero‐Prisno

**Affiliations:** ^1^ Faculty of Postgraduate Medicine Khesar Gyalpo University of Medical Sciences of Bhutan Thimphu Bhutan; ^2^ Department of Internal Medicine Central Regional Referral Hospital Gelegphu Bhutan; ^3^ World Health Organization South‐East Asia Regional Office New Delhi India; ^4^ Department of Global Health and Development London School of Hygiene and Tropical Medicine London UK

**Keywords:** Bhutan, health equity, health policy, healthcare financing, out‐of‐pocket expenditures, universal healthcare

## Abstract

Bhutan has a free healthcare system that covers almost 90% of the population within 2 h of travel distance. The country has achieved remarkable success in many public health indicators despite the chronic shortage of financial resources and trained manpower. However, there are many aspects of health inequities in the government's health policies, programmes and health services. The Khesar Gyalpo University of Medical Sciences of Bhutan, Thimphu in 2020 hosted a policy dialogue on health equity in the context of the Bhutanese health system. With changing demographics and socioeconomic conditions, some of the factors that earlier determined an equitable distribution of services and resources are no longer relevant now. The referral system is easily bypassed not only because the patients have easy access to transportation to tertiary hospitals, but because of frequent interruptions in the service and the non‐availability of doctors in the district and general hospitals. The role of the private sector is restricted to a few diagnostic services while there is an apparent adequate spending capacity of consumers. There is an important component of out‐of‐pocket expenditure and catastrophic health expenditure in patients seeking treatments outside the country. The current health policies and strategic plans for the future lack a measure of health equity. We recommend conscientious assessment of health inequities in the current system and introducing policies and programmes to prevent the worsening of such inequities.

## Graphical abstract

Men dancing after an arrow hits the target during a play of archery match, the national sport of Bhutan.

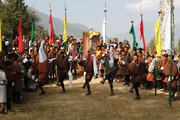

## INTRODUCTION

Health equity is a crucial component of social justice and human development; however, it is often forgotten in the assessment of national policies, implementation of health programmes and the delivery of health services. Equity in health is the absence of systematic disparities in health between groups with different levels of underlying social advantage/disadvantage, such as wealth, power or prestige [1]. The World Health Organization's Commission on Social Determinants of Health in 2008 reported on the factors that influence equity in health policies, programmes and services—daily living conditions, social and economic conditions; health finances and human resources; and socio‐political systems and overall governance of health systems [2]. While there have been debates on what defines health equity, the Commission's report calls for attention to reduce the inequalities or disparities in one's ability to achieve one's right to health—a state of complete physical, mental and social well‐being and not merely the absence of disease or infirmity. While some of the determinants of health equity may be common across health systems and countries, the level of inequities/disparities and their differential contribution to the problem need to be studied and addressed within the specific socio‐economic, political and cultural contexts of health systems for that particular country.

Bhutan is situated in the eastern Himalayas and is home to a population of 0.72 million. In Bhutan, all aspects of modern healthcare are provided free of cost by the government [3]. The system is a pro‐poor design as evidenced by benefit incidence or public benefits of health expenditure in 2018 across outpatient, inpatient, obstetrics and primary healthcare services [4]. However, this analysis also reported on the unequal distribution of benefit incidence with people in rural areas availing substantially lower shares of all types of healthcare.

Currently, there are no direct assessments of health equity/inequity in health services, financing and health policy formulation for Bhutan. The Khesar Gyalpo University of Medical Sciences of Bhutan, Thimphu, hosted a policy dialogue titled “Health Equity in Bhutan: understanding access and improved health outcomes” on 23 October 2020. This was a blended virtual and in‐person conference involving expert speakers from the academe, the government and the World Health Organization and participants that included health workers, journalists and members of government agencies and civil society organizations. The expert panel presentations were engaging and were followed by a round of comments and sharing of experiences of inequities and a wide‐range of questions and recommendtions. The policy dialogue generated rather many unanswered questions, and we followed them up through multiple rounds of online and offline discussions. In this article, we provide our assessment and interpretation of health inequity status in Bhutan, some of its determinants and the way forward in addressing these inequities.

## HEALTHCARE SYSTEM IN BHUTAN

The health system in Bhutan adopted the primary healthcare approach after becoming a signatory to the Alma‐Ata Declaration in 1978 [3, 5]. In 2022, Bhutan had a three‐tiered health care system with 179 Primary Health Centres, 53 sub‐posts and 555 outreach clinics at the primary level, 49 district and general hospitals at the secondary level and three referral hospitals at the tertiary level [6]. The three‐tiered system is designed to streamline the referral system, for the judicious allocation of human resources and delivery of level‐appropriate services.

In Bhutan, the state prioritizes the well‐being of its subjects and is mandated by the Constitution to “provide free access to basic public health services in both modern and traditional medicines” and is guided by the overall principle of Gross National Happiness (GNH) [7]. Indeed, health services at all levels are provided free of cost including treatment in empanelled hospitals outside the country for high‐end therapies.

The country has achieved remarkable successes in public health sectors such as maternal and child health, vaccine‐preventable diseases in children and women, relative control of communicable diseases such as leprosy, improvement in access to safe drinking water and sanitation, reduction in diarrhoeal diseases in children and an overall increase in life‐expectancy to 70.2 years (Table 1) [8]. The health infrastructure covers more than 90% of the population within 2 h of travel distance, 99.4% of the population have access to improved drinking water services and 95.2% have access to improved sanitation facilities [9, 10]. There are no overt health inequities or disparities in terms of conventional comparisons such as the percentage coverage of the health network, staffing and health financing and primary healthcare indicators. However, analyses of health service delivery parameters by the panelists and an engaging question‐answer session with the participants at the Policy Dialogue brought forth the areas of health inequities that require careful review and attention before allowing further widening of the health gap.

**TABLE 1:** The status of selected national indicators of health development in Bhutan, 2021 [6]

Sustainable development goal/equivalent national indicator	Current status
Maternal mortality ratio	89 per 100,000 live births
Deliveries attended by skilled health worker	98.9%
Institutional delivery	98.1%
Under‐five mortality rate	34.1 per 1000 live births
Neonatal mortality	21 per 1000 live births
Total new HIV cases	56
Total TB cases detected	858
Indigenous malaria incidence	0.06 per 1000 population
Total morbidity cases with acute hepatitis B	11.5 per 100,000 population
Suicide rate	12.7 per 100,000 population
Population 15–75 years who ever used drugs/substance	2.1%
Death rate due to road traffic injuries	73 per 100,000 population
Prevalence of contraceptive use among 15–49 years	65.6%
Unmet need for family planning	11.7%
Women 15–49 years who knew at least one method of contraception	96.3%
Adolescent (15–19 years) birth rate	14.2 per 1000 women in that aged 15–19 years
Percentage of population within 2 h to the nearest health facility	87.7
Percentage of death due to illness	80.7

## POPULATION DEMOGRAPHICS AND ACCESSIBILITY TO HEALTH SERVICES

Bhutan's population is scattered over mountainous terrain where physical access to health centres is challenging because of arduous distances and lack of transportation means. Between 2005 and 2011, the government made major investments in the expansion of health infrastructure across the country which now covers 87.7% of the population within 2 h of travel distance from the nearest health centre [3, 10]. While the government has taken health service centres into the villages, there has been a steady increase in the migration of population by 7.1% (or 80,563 persons) between 2005 and 2017 [11]. During this period, districts in the western region such as Thimphu and Phuentsholing municipality have seen a net increase in migrant population while districts in the eastern region such as Zhemgang, Lhuentse, and Trashigang have seen a net loss of population [11]. This has resulted in significant changes in the demographic composition of the elderly population aged 65 years and older. The rural districts such as Pema Gatshel and Zhemgang have more percentage of the elderly population than the national average [11].

Health infrastructure planning and distribution is planned based on catchment population but rural‐urban migration of people has resulted in overcrowding in hospitals in urban centres while some health facilities in rural areas have remained underutilized. Centres like Gasa and Haa have reported respectively 13 and 20 admissions per 1000 population in 2020 and 8 and 22 admissions per 1000 population in 2021 [6, 12]. Patients from districts located within shorter distances from referral centres directly present to these  centres , bypassing the traditional referral system. As a result, there is overcrowding at the referral hospitals for similar services available at district hospitals resulting in long waiting hours for consultation with doctors, for investigations such as neuroimaging and surgeries. Table 2 shows the combined in‐patient admissions in the hospitals across 20 districts that show skewed patient load in referral centres.

**TABLE 2:** The population of districts and the total number of all hospital admissions in Bhutan, 2021 and 2022

		Year 2020		Year 2021	
Dzongkhag/districts	Population^b^	Hospital admissions^c^	In‐patients per 1000 population	Hospital admissions^d^	In‐patients per 1000 population
Bumthang	17,820	811	46	793	45
Chhukha	68,966	4104	60	3888	56
Dagana	24,965	1197	48	1337	54
Gasa	3952	50	13	32	8
Haa	13,655	268	20	300	22
Lhuentse	14,437	706	49	587	41
Monggar^a^	37,150	4648	125	4190	113
Paro	46,316	2335	50	2063	45
Pema Gatshel	23,632	1106	47	1002	42
Punakha	28,740	1649	57	1265	44
Samdrup Jongkhar	35,079	1779	51	1833	52
Samtse	62,590	3351	54	3285	52
Sarpang^a^	46,004	2907	63	3662	80
Thimphu^a^	138,736	16,524	119	13,924	100
Trashigang	45,518	3456	76	3027	67
Trashi Yangtse	17,300	2122	123	906	52
Trongsa	19,960	617	31	512	26
Tsirang	22,376	2504	112	1300	58
Wangdue Phodrang	42,186	2601	62	3205	76
Zhemgang	17,763	615	35	552	31
**Total**	**727,145**	**53,350**	**62 (mean)**	**47,663**	**53 (mean)**

^a^The referral hospitals are located in three districts: Thimphu, Monggar and Sarpang.

^b^Population and Housing Census of Bhutan 2017, National Statistics Bureau, Bhutan [11].

^c^Annual Health Bulletin 2021, Ministry of Health, Bhutan [10].

^d^Annual Health Bulletin 2022, Ministry of Health, Bhutan [6].

While road networks have improved in many places, factors other than the availability of transportation, cause difficulties in accessing healthcare. For example, women in urban settlements deliberately delayed their first antenatal visit in order to reduce the total number of hospital visits to save costs of travel [13].

In the three‐tiered health system, specialist consultations and select health services such as maternal exercise programme during the antenatal period, specialized physiotherapy and rehabilitation services [14] and haemodialysis are available only in selected hospitals requiring the patients to either travel to these hospitals or migrate and live near such centres. For many patients with chronic conditions, frequent commute and transportation are not affordable [15]. In such situations, civil society organizations such as the Bhutan Kidney Foundation and Bhutan Cancer Society have stepped in to provide support.

## IS THERE A NEED FOR EQUITABLE ALLOCATION OF HEALTH HUMAN RESOURCES?

In 2021, there were 354 doctors (143 general doctors, 136 specialists, 75 dental surgeons) and 1608 nurses in the country with a ratio of 4.6 doctors and 21.1 nurses in 10,000 population [6], somewhat lower than the World Health Organization's regional threshold of 22.8 [3]. The current staff deployment is driven by infrastructure‐based allocation with a traditional norm of allocating two to three health assistants or nurses for each primary health care centre. This trend of staff deployment does not match the patient load and ground‐level requirements and leads to an apparent underutilization in some centres (as shown in Table 2) and overburdening with high patient load in others.

Human resource planning and deployment need to consider dynamic factors such as transfer of staff, staff on study leave and those resigning/retiring from service. Often, there are gaps of many months to years when a replacement doctor is posted in hospitals during which people have to travel to higher health centres due to discontinuity of services. Among those who cannot afford self‐referral to higher centres, there is a definite delay in diagnosis and treatment of disease conditions leading to an overall increase in the cost of treatment.

In 2020–2021, the health human resource pool and its performance were tested when the government deployed doctors, nurses and technicians in COVID‐19 management. To fill the gap, the government recalled 46 doctors who were undergoing postgraduate training outside the country and also involved student nurses and technicians from the Khesar Gyalpo University of Medical Sciences of Bhutan in the pandemic efforts and in providing an uninterrupted delivery of essential services [16]. The country lacks a medical college and all its undergraduate medical students and dental surgeons are trained outside. There is an urgent need for an assessment of current and future health human resource gaps in order to provide quality services.

## HIDDEN COSTS AND OUT‐OF‐POCKET PAYMENTS IN A FREE HEALTHCARE SYSTEM

Healthcare is financed by the Royal Government of Bhutan with a total health expenditure of Nu 8.7 billion (US dollar 117 million), constituting 4.5% of the gross domestic product in 2019–2020 [10]. The total health expenditure ranged between 3.6% and 5.3% of the gross domestic product between 2005 and 2014 [10].

The government provides free healthcare but out‐of‐pocket expenditure as a percentage of total health expenditure has ranged between 11% and 12% between 2010 and 2014 [3]. In 2017, a patient's family spent an average of Nu 2304 (US dollar 31) on treatment and services during a period of hospitalization. Around 58% of the expenditure was spent on religious rituals, 25% on transportation charges and 7% on the purchase of medicine and health accessories [17]. Such out‐of‐pocket payment is on the rise with an increase in cost‐of‐living and inflation, especially in the bigger towns where referral hospitals are located. The out‐of‐pocket payment is even more significant when patients are referred for health services in countries outside Bhutan. Events of catastrophic health expenditure have also been reported forcing patient families to seek public support and donations of millions of ngultrums (Nu 1 million = US dollar 12,500 as of September 2022) to fund the treatment services.

The government's out‐country referral of cases requiring high‐cost treatments is based on strict selection criteria that are often based on minimizing government expenditure. Given that the government's health expenditure has to prioritize the common good, select cases requiring investigational therapeutics involving high cost and those with less likelihood of survival have not received government support in out‐country referral and treatment. This calls into question the limits of healthcare services that can be provided and balances the sustainability of funding a free healthcare system.

Similarly, within the free healthcare system, there is a set of medical services such as dentures, composite restoration, crowns and bridges, dental scaling and orthodontic treatment with installation of braces require payment even in the government hospitals as these are deemed non‐essential health services. In many hospitals, patients can choose to pay for single‐room cabin services during their hospital stays which have facilities for family members to accompany the patient and have separate bathroom facilities. Although these services are provided at subsidized rates, there are many who are unable to afford such facilities and are required to share common bathrooms and lack space for their family members or patient attendants.

## HOW ROBUST IS THE GATEKEEPING FUNCTION OF THE REFERRAL PATHWAYS?

Bhutan's three‐tiered health system is designed for proper care coordination and referral of patients with free ambulance services. Cases requiring specialist services are referred to designated referral centres while cases requiring supportive or palliative care may be down‐graded from specialist to general hospitals. However, this referral system is bypassed by many and patients present to the tertiary care centre of their choice. The question is what happens to those who are unable to jump the queue? For example, a patient presenting with a headache gets to consult a specialist and get neuroimaging at a tertiary hospital while a similar patient in a district hospital or a primary health care centre is seen by a general doctor and does not have access to investigations.

One of the reasons for bypassing of referral system is when there are interruptions of services such as blood tests, X‐rays or ultrasonography. There are many instances of the unavailability of services when there is a machine breakdown or when technicians or doctors are unavailable to operate them. When CT scanning was not available at the Jigme Dorji Wangchuck National Referral Hospital for many months at a stretch, patients and family members had to travel across the border to India. This incurs not only additional financial costs but is a major logistic challenge for many families and patients, especially those who lack social support, leading to loss‐to‐follow‐up and attrition from care. Therefore, in order to provide continued health services within the country, regional centres with backup machines and technicians are recommended.

Many patients often present at hospitals with higher levels because of the belief that the quality of care is better. The Ministry of Health has initiated mobile camps where a group of specialists visits the villages providing screening tests. However, most of these camps remain a one‐point contact with no arrangements for follow‐up specialist care. The Ministry of Health has been exploring means to upscale the standards of care provided through the primary healthcare system. The PEN HEARTS initiative project in the districts of Punakha, Wangdue Phodrang, Tsirang and Zhemgang demonstrated that appropriate training of health professionals at the grassroots and developing treatment guidelines helped achieve standards of care in hypertension and diabetes through the health assistants at the primary health centres.

It must also be noted that bypassing the referral system may be counterproductive in terms of patient outcomes. For example, all institutional deliveries in Bhutan are attended by skilled professionals—health assistants, midwives and doctors at the primary and second‐tier hospitals, and by obstetricians at the seven obstetric centres [18]. Many women with no high‐risk pregnancy bypass the referral channel and present to these obstetric centres for delivery. In the absence of national standards and guidelines on making decisions on caesarean deliveries, these women end up with more likelihood of delivering babies by caesarean section at centres where an obstetrician is available. Bhutan has an increasing rate of caesarean deliveries which may be counterproductive towards the 2030 Sustainable Development Goal agenda [18].

## PRIVATE PARTICIPATION IN HEALTHCARE SERVICE DELIVERY

The private sector has been playing an important role in health service delivery. The private sector is involved in the health screening of expatriates coming into the country and the delivery of equipment, lab reagents and drugs to government hospitals. They also are actively involved in the multi‐sectoral task force in every district in advocacy for HIV/AIDS, safe sex and family planning [3]. In large townships, the private sector also provides diagnostic facilities such as laboratory tests, endoscopy and sonography. The private diagnostic centres are popular among people given their people‐friendly timings, efficient service delivery and availability of services such as dental and dermatological procedures that are otherwise classified as non‐essential services in the government sector. While no formal studies are available, there is apparent consumer ability to pay for the healthcare service of their choice. With improving financial capacity to pay for health services, it is equally important for users to be given adequate choices. Private hospitals may be able to cater to the specific needs of sections of the population such as personalized birthing experiences, dental services, dermatological and cosmetic services and help reduce government spending.

However, there is widespread apprehension about allowing the private sector in healthcare services that such a move might increase the gap between the rich and poor. There is minimal involvement of the health insurance system mainly because all levels of healthcare are provided free of cost by the government. The government on the other hand is facing difficulties in finding a sustainable solution for health financing in the background of increasing expenditure and increasing health service expectations from the citizens.

## OPERATIONALIZATION OF HEALTH EQUITY

**FIGURE 1:**
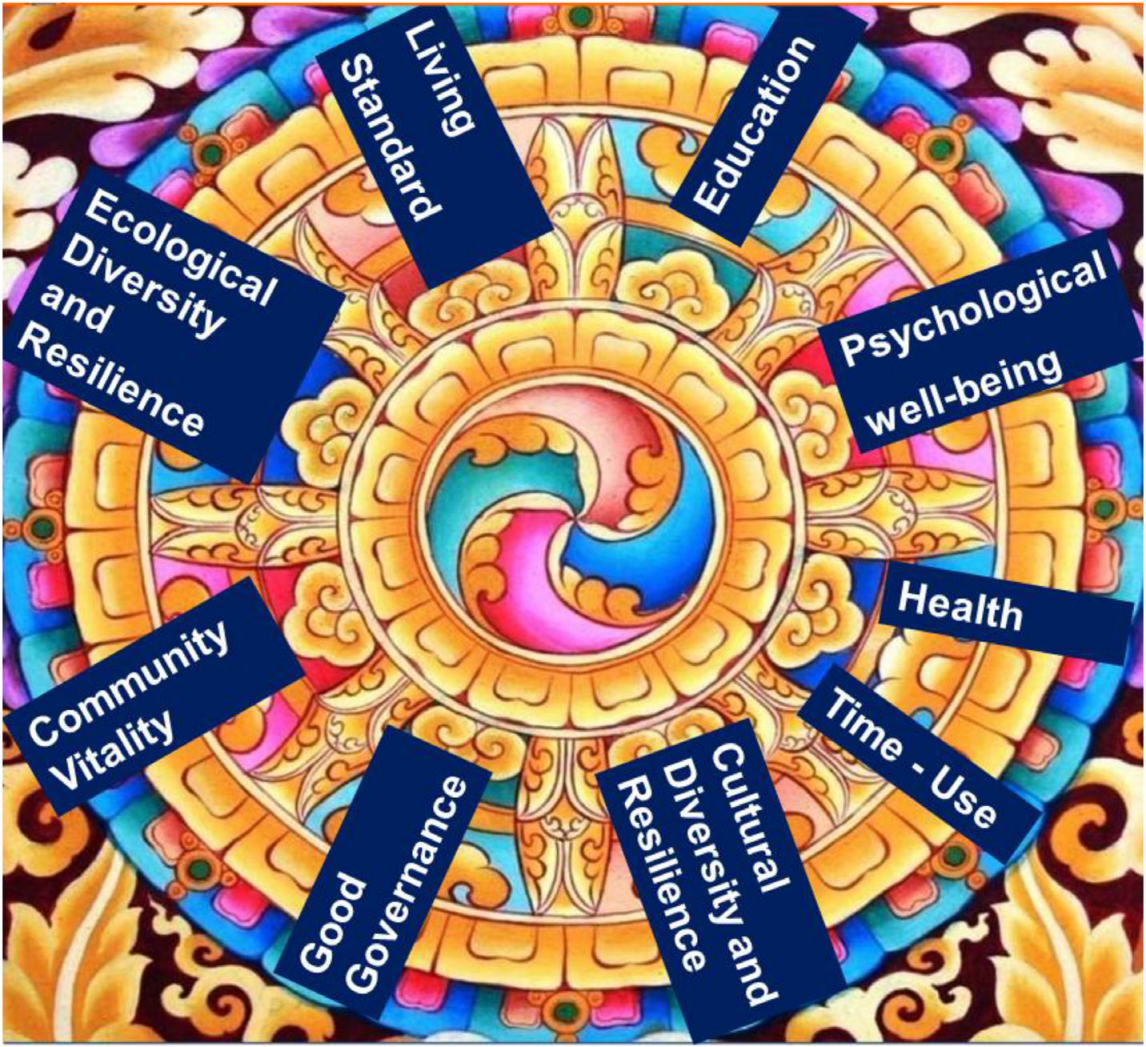
Health is one of the domains of the Gross National Happiness Index. Health is described under the themes of self‐reported health status, number of healthy days, disability and mental health [19]

Health equity involves normative judgement of fairness which is often difficult to measure. In Bhutan, the government pursues GNH as a guiding principle for inclusive and sustainable development in all spheres of human life [7]. Health is one of the nine domains of the composite GNH Index (Figure 1) [19]. However, current approaches to the measurement of the GNH index do not include systematic data on health equity. Hence, the long‐term impacts of the current health and development policies and programmes on health equity are still not well understood in the country.

Similar to how policies are assessed for other standards of fairness, there is a need to undertake conscientious health equity assessments in the health policies and programmes. Equity assessment begins at the stage of drafting the policy, obtaining legislative support and implementing such policies in coordination with agencies outside the Ministry of Health. Since the health sector is fully funded by the government, legislators play an important role in the allocation and approval of the budget for the health sector against the backdrop of competing interests for government spending highlighting the need for health equity sensitization at the highest levels. A health equity indicator tool may be developed to assess and score policies in terms of health equity at different levels of its formulation; this indicator tool may be designed and used in similar ways as the GNH indicator tool which is in current use in the country.

Health policies and health outcomes are achieved through a well‐functioning health system with good leadership. The Ministry of Health currently lacks experts in many disciplines including those in health equity assessment. Health managers in the field of health services, health promotion, epidemiology and disease control, disease management, rehabilitation and health human resource managers should be sensitized on the importance of health equity assessment at the level of their work.

## CONCLUSIONS

Health equity in Bhutan's free healthcare system has not been documented adequately before. While the health system has achieved much success in many areas of public health and clinical health indicators, there are inequities in health service delivery, human resource planning and deployment, health policy formulation and health financing. These inequities must not be discounted as the country lacks a proper assessment of the health equity impacts of its present health policies. There is a need to sensitise the policy makers and health managers across all levels on the need to reduce inequity gaps in the health system.

## AUTHOR CONTRIBUTIONS

All authors were involved in the conception of this manuscript. Karma Tenzin, Thinley Dorji and Don Eliseo Lucero‐Prisno III drafted the manuscript. All authors were involved in critical review and have given final approval for publication of the materials.

## CONFLICT OF INTEREST

TD and DELP are editorial board members of the journal. They were excluded and blinded from all stages of peer review of this manuscript.

## ETHICS STATEMENT

Not applicable.

## CONSENT FOR PUBLICATION

Not applicable.

## Data Availability

All relevant data sources are cited in this article.
